# A mixed‐methods analysis of younger adults' perceptions of asthma, self‐management, and preventive care: "This isn't helping me none"

**DOI:** 10.1111/cea.13751

**Published:** 2020-10-18

**Authors:** Jennifer R. Mammen, Kelsey Turgeon, Ashley Philibert, Judith D. Schoonmaker, James Java, Jill Halterman, Marc N. Berliant, Amber Crowley, Marina Reznik, Jonathan M. Feldman, Robert J. Fortuna, Kimberly Arcoleo

**Affiliations:** ^1^ College of Nursing University of Rhode Island Kingston RI USA; ^2^ University of Rochester School of Nursing Rochester NY USA; ^3^ Department of Biostatistics and Computational Biology University of Rochester Rochester NY USA; ^4^ Department of Pediatrics University of Rochester School of Medicine Rochester NY USA; ^5^ Department of Internal Medicine University of Rochester School of Medicine Rochester NY USA; ^6^ Department of Pediatrics Division of Academic General Pediatrics Albert Einstein College of Medicine Children’s Hospital at Montefiore Bronx NY USA; ^7^ Ferkauf Graduate School of Psychology Yeshiva University Bronx NY USA

**Keywords:** asthma, self‐management, young adult

## Abstract

**Background:**

Young adults (ages 18‐44) have increased emergency department use for asthma and poor adherence to medications. The objective of this mixed‐methods study was to understand experiences with and approaches to managing asthma, of which little is known in this age group.

**Methods:**

Surveys (Asthma Control Questionnaire, Asthma Quality of Life Questionnaire) and 1:1 semi‐structured interviews were used to explore experiences with asthma, symptoms, self‐management behaviours, and relationship to asthma control and quality of life. Qualitative data were analysed using content analysis techniques. Descriptive statistics and bivariate correlations were used to examine distributive characteristics and associations between variables.

**Results:**

Forty urban adults participated (mean age 32.7 ± 6.2, 1σ). Coughing was reported nearly 46% more often than wheezing, with 42.5% (17/40) coughing until the point of vomiting most days. Most participants delayed using medication for symptoms due to misperceptions about inhalers. Higher symptom frequency and worse asthma control were associated with greater use of non‐pharmacologic symptom management strategies (r = 0.645, *P* < .001; r = 0.360, *P* = .022, respectively). Five themes were identified regarding young adults experiences with asthma: (1) having asthma means being limited and missing out on life; (2) health care for asthma is burdensome, and other things are more important; (3) there is not enough personal benefit in medical interactions to make preventive care worthwhile; (4) there are insufficient support and education about asthma for adults; and (5) people normalize chronic symptoms over time and find ways of coping that fit with their lifestyle.

**Conclusions and Clinical Relevance:**

Young adults may tolerate symptoms without using quick‐relief medication or seeking preventive care. Increasing engagement with preventive services will require decreasing perceived burdens and increasing the personal benefits of care. Evaluating for non‐pharmacologic approaches to managing symptoms and asthma‐related coughing may identify uncontrolled asthma. Enhanced training for clinicians in patient‐centric asthma care may be needed.

## 1 INTRODUCTION

Despite the availability of effective treatment for asthma, the majority of young adults with asthma have persistently uncontrolled disease (ages 18‐44 years, >58% uncontrolled asthma in 2016, US population data,).^1^ Adherence to controller medications has been estimated at 14.5%‐23.5%,^2^ with rates of emergency department use for asthma exacerbations are higher than either younger or older age groups.^3^, ^4^ These high‐risk hallmarks suggest increased burden of asthma and an urgent need to improve outcomes in this age group.

Albeit having transparently poor asthma outcomes, little is known about asthma self‐management in young adults.^5^, ^6^, ^7^, ^8^ To date, most research in this area has been derived from paediatric and general/older adult populations.^9^, ^10^, ^11^ While there is some evidence that adolescent patterns of self‐management (eg poor symptom recognition and declining medication adherence) extend into adulthood and contribute to worsening clinical outcomes,^12^, ^13^ other research indicates that young adults might have unique needs and challenges.^14^, ^15^ Lower‐income urban young adults may be particularly at risk, having poorer health literacy, fewer resources, and decreased access to consistent high‐quality care.^15^, ^16^, ^17^ However, information about this population is scarce.^5^, ^6^, ^18^, ^19^

The ultimate goal of health care and related research is to improve outcomes and enable people to live well, unimpeded by disease. By extension, this means helping patients develop, implement, and maintain effective asthma self‐management strategies, which are, in turn, contingent upon the willingness and ability of individuals to perform specific self‐management tasks.^20^, ^21^ Therefore, an important step in optimizing self‐management is to *first* understand how people manage their asthma and why they do what they do.^9^, ^22^ This knowledge is important for both clinicians and researchers, as oversight of key factors could impede ability to deliver care or devise effective interventions. Thus, the purpose of this study was to explore young adults perceptions and experiences of asthma, usual approaches to asthma management, and underlying rationales for behaviours.

## 2 METHODS

This was a mixed‐methods observational study, including quantitative surveys, lung function, and 1:1 qualitative descriptive interviews. The study was approved by the University of Rochester as part of a broader interventional study for young urban adult smartphone users (NCT03648203, Ethics committee review 10 October 2017, RSRB67900).^23^, ^24^, ^25^

### 2.1 Setting, sample

Forty patients were recruited from a safety‐net resident‐run primary care clinic in Western NY. This type of practice provides care to many lower socio‐economic status individuals who might otherwise not have access to consistent primary care. Eligible participants were the following: (1) English speaking, (2) with persistent asthma by Expert Panel Report‐3 (EPR3) criteria,^26^ (3) aged 18‐44 years, (4) smartphone users, (5) not pregnant, and (6) without confounding respiratory or cardiac diagnoses. A randomized roster of all patients aged 18‐44 with asthma was generated for the participating practice using the electronic medical record (years 2018‐2019). Letters were mailed to the first 140 patients on the randomized list notifying of intent to contact and offering patients a chance to "opt‐out," but none elected to do so. This was followed by a screening phone call to consecutively listed patients 2‐weeks later. Of the first 65 individuals reached by phone, 55 were eligible. Nine of these declined (unstated reasons), 6 were lost to contact, and 40 (72.7%) completed informed consent and participated in the study.

### 2.2 Data collection and measures

All data were collected by a trained research assistant (RA) during a single home visit.

#### 2.2.1 Demographic and asthma surveys

Surveys were used to gather data on asthma knowledge, symptoms, perceptions of severity and control, emergency department use, satisfaction with asthma care, and demographics. Frequency of emergency use was measured by self‐report to capture in‐ and out‐of‐network visits in the preceding year. All participants completed surveys via personal smartphone (paper copies were available but not utilized).

#### 2.2.2 Severity and control

Asthma control was measured using the self‐administered version of the paper‐based *Asthma Control Questionnaire* (ACQ). The ACQ is a 7‐item Likert scale survey with item and total scores ranging from 0 to 6. Cronbach alpha is ≥ 0.82, and test‐retest reliability is ≥ 0.75.^27^ Lower scores indicate better asthma control, and a score of 1.5 has a positive predictive value of 0.88 for uncontrolled asthma.^28^ Asthma severity and control according to the National Heart Lung and Blood Institute EPR3 guidelines were determined by symptom frequency, nocturnal awakening, activity limitations, and use of short‐acting beta‐agonist (SABA).^29^

#### 2.2.3 Forced expiratory volume (FEV1)

Forced expiratory volume was measured during the in‐home visit via Microlife Peak Flow Meter (PFM)^30^ which has validated accuracy to within 5% of the reading or ± 0.1 litres. Data were collected by the study RA, and patients were trained in metre use and maximal effort prior to measurement. Percentage predicted (FEV_1%pred_) was determined by the National Health and Nutrition Examination Survey (NHANES) criteria.^31^

#### 2.2.4 Quality of life (QoL)

Quality of life was measured using the self‐administered version of the paper‐based Asthma Quality of Life Questionnaire (AQLQ), which measures physical and emotional impact of disease. The AQLQ is a 32‐item Likert scale survey with item and total scores ranging from 1 to 7. Cronbach alpha is ≥ 0.90, and test‐retest reliability is ≥ 0.95.^32^, ^33^ Higher scores indicate better quality of life.

#### 2.2.5 Qualitative interviews

Following the surveys, each participant engaged in a private 1:1 semi‐structured audio‐recorded interview (average 43 minutes, range 26‐94) with a trained research assistant unknown to participants (JS; older, female, White, with social work background) using scaffolded interview questions derived from the Asthma Self‐management Model^21^ (Box 1). All participants were aware of the purpose of the study. Questions were designed to explore experiences with asthma, perceptions of asthma, and approaches to self‐management, along with underlying rationales for self‐management behaviours. Field notes were recorded for each interview and shared with the research team prior to data analysis.

**Sample interview questions**

1. Talk to me about your asthma. Can you tell me about your experiences?
  - What are your experiences getting healthcare for asthma?
  - What have you been taught about your asthma?
  - What are the biggest challenges in managing or treating your asthma?
2. How do you feel about your current asthma management?
  - What works and/or doesn't work for you?
3. What kinds of things help you manage your asthma better?
4. What does having well controlled asthma mean to you?
5. Tell me about your asthma medications:
  - What is a control inhaler to you? What is a rescue inhaler?
  - How do you feel about taking daily control medication (__)? How do you feel about taking rescue medication (__)? Describe how/when/where you use it or not use it. How do you decide?
6. With card‐sort activity, after mapping symptoms/responses: Think about the last time you had asthma symptoms. Describe exactly what you experienced and what you did to manage the symptoms.
  - What kinds of things do you usually do to manage your asthma? (medical or non‐medical)
  - Explain where and why you do each thing.
  - When you take medication, how does it work?

#### 2.2.6 Symptom/response card sorting

**Figure 1:**
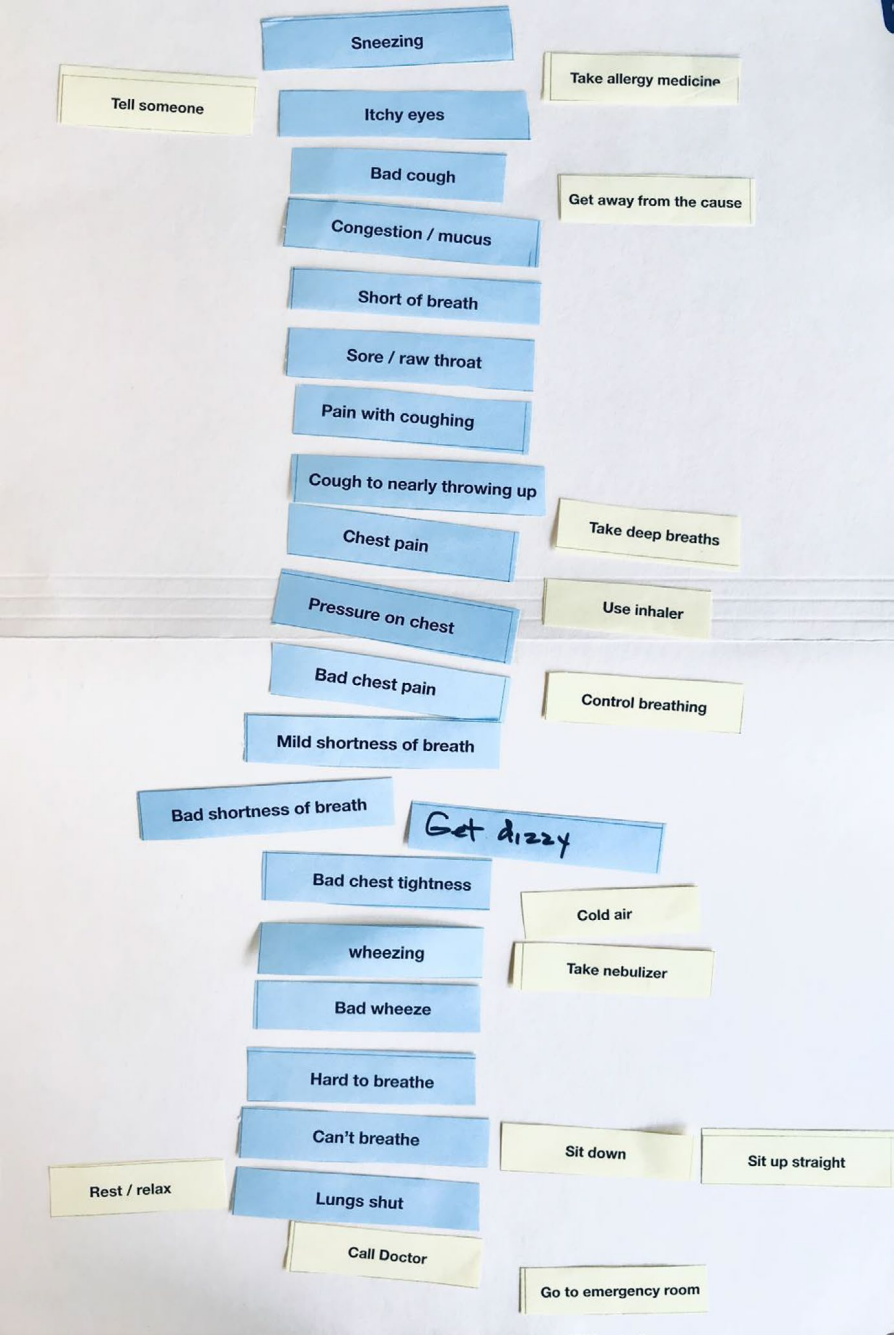
Image of participant card sort showing perceived asthma symptoms and specific self‐management responses to symptoms (top‐down order of occurrence; P9)

Symptom/response card sorting^34^, ^35^ was used to map each participant's usual symptom pattern and self‐management responses and to elicit detailed information about experiences along with rationale for behaviours. For this activity, participants first identified their personal symptoms and self‐management responses via a checklist developed in prior research.^35^, ^36^ They were then given printed cards of the selected items and asked to arrange their symptoms/responses in order of occurrence, modifying words and cards as needed to create a visual depiction of their usual symptom/response pathway (Figure 1). Symptoms or responses that occurred more than once were quantitatively represented by additional cards. For example, participants who used an inhaler twice during their symptom pathway would include two inhaler cards in the map, next to the symptoms for which the inhaler would be used. Participants then described and discussed their symptom experiences, usual responses to symptoms, and rationales for behaviours, along with any commonly occurring situational differences in response.

### 2.3 Data analysis

Qualitative data analysis occurred contiguously with data collection. Enrolment exceeded data saturation, and no new codes were identified after the 35th participant. Transcribed interviews and card sorts were analysed by JM, JD, KT, and AP using Nvivo12 and a qualitative descriptive consensus coding approach.^37^ Open coding was performed first. Content analysis was used to analyse images and interviews for symptom type, frequency, severity, and patterns of symptoms/self‐management responses, including use of pharmacologic and non‐pharmacologic symptom management strategies.^38^ Frequencies were calculated as the percentage of participants who experienced a particular symptom *and* the total number of instances that symptom was mentioned during interviews, as word frequency is a proxy indicator of importance to the individual.^39^, ^40^ Lastly, data and codes were mapped using Xmind to develop thematic patterns and synthesized to define key concepts.^41^, ^42^ Steps to enhance validity were the following: (1) structured memos; (2) member checking; and (3) peer debriefing, and (4) use of participant identifiers for quotations in the manuscript, including race, sex, and age.^43^ Statistical analyses were performed using SPSS 25. Descriptive statistics were used to examine distributional characteristics of the data. Bivariate correlations were used to examine associations between linear variables including asthma control (ACQ), quality of life (AQLQ), emergency care, symptoms, and self‐management strategies.

## 3 RESULTS

Demographics are presented in Table 1. Participants were predominantly lower socio‐economic status, of minority ethnicity, with uncontrolled asthma (82.5% uncontrolled by ACQ, 100% uncontrolled by EPR3 classification).

**Table 1:** Demographics and asthma characteristics (N = 40)

Demographic characteristics	N	%
Sex (female)	28	70%
Race/ethnicity		
Black	21	52.5%
White	8	20%
Hispanic/Latino	5	12.5%
Multiracial	5	12.5%
Asian	1	2.5%
Insurance (public)	30	75%

	Mean	SD
Age (years)	32.75	6.18
Household income (USD per year)	$29,420	$14,300

Asthma characteristics	N	%
Asthma Severity (EPR3)^a^		
Mild	5	12.5%
Moderate	24	60%
Severe	11	27.5%
Asthma control (EPR3)		
Well controlled	0	‐
Not well controlled	20	50%
Very poorly controlled	20	50%
Asthma control (ACQ)^b^		
Well controlled	7	17.5%
Not well controlled	33	82.5%
Asthma control (participant perceived)		
Well controlled	8	20%
Not well controlled	20	50%
Very poorly controlled	12	30%
Current quick‐relief medication use		
None most days	12	29.3%
1‐2 puffs most days	7	17.1%
3‐4 puffs most days	13	31.7%
5‐8 puffs most days	6	14.6%
9‐12 puffs most days	0	‐
13‐16 puffs most days	0	‐
> 16 puffs most days	2	4.9%
Current controller medication	21	52.5%
Emergency visit in past 12 months	15	37.5%

	Mean	SD
Baseline ACQ score^b^	2.27	0.99
Baseline AQLQ score^c^	4.27	1.18
Baseline FEV1% predicted	80.55	14.57
Years diagnosed with asthma	19.55	10.17

Note

^a^ Expert Panel Report 3, National Heart Lung and Blood Institute (2007).

^b^ ACQ = Asthma Control Questionnaire (range 0‐6), lower scores represent better asthma control, a score of ≥ 1.5 has 88% positive predictive value for uncontrolled asthma.

^c^ AQLQ = Asthma Quality of Life Questionnaire (range 1‐7), higher scores represent better quality of life

Participants identified an average of 11 different symptoms (SD = 4.4) and 10 self‐management responses daily (SD = 5) on the surveys. Two‐thirds (25/40) reported having severe symptoms on a regular basis (eg difficulty speaking, cough to point of vomiting, severe chest pain most days or daily^44^). For example:

P4: A typical day, it’s like breathing through like a tiny hole…I feel like the elephant's sitting my chest. (Black, female, age 29)

Table 2 shows symptoms by frequency in interview transcripts versus card‐sort images, including the *percentage* of participants who reported each symptom and the total number of *instances* a symptom occurred in each modality (interview transcripts vs. card‐sort images). For example, coughing was reported by 90% of participants, but total instances of coughing (n = 419) were 46% greater than total instances of wheezing (n = 287), indicating that those with asthma‐related coughing talked about coughing far more often than they talked about wheezing. Chest pain/pressure was also discussed more commonly than chest tightness (2:1), and bothersome throat‐clearing was frequently reported. Nearly 43% of participants (17/40) identified coughing to the point of vomiting regularly, as seen here:

P26: "every morning I wake up, I can’t breathe, I start coughing hard, then throwing up…I keep a bucket beside my bed…” (Black, Female, age 40)

**Table 2:** Self‐reported frequencies of symptoms and self‐management responses

	Card sort	Interview	Card sort	Interview
	% Participants		No. Instances	
Symptoms				
Wheezing	100%	97.5%	64	287
Shortness of breath	95%	100%	63	234
Coughing	90%	95%	75	419
Chest pain or pressure	70%	80%	73	248
Struggling to breathe	67.5%	90%	59	265
Chest tightness	62.5%	82.5%	33	130
Throat symptoms	50%	75%	27	111
Chest congestion	52.5%	52.5%	21	48
Allergy symptoms	47.5%	57.5%	24	74
Coughing to point of vomiting/vomiting	42.5%	45%	23	59
Responses (self‐management strategy)				
Use any non‐pharmacologic approach	100%	100%	214	825
Getting a drink	70%	77.5%	50	244
Rest, slow down, sit down	65%	100%	32	258
Control breathing	67.5%	62.5%	50	104
Calm down/relax	32.5%	80%	32	102
Wait/tough it out	25%	77.5%	12	117
Use steam/shower/cool air	37.5%	37.5%	10	45
Trigger avoidance	37.5%	35%	28	22
Use any asthma medication (inhaler/nebulizer)	95%	100%	85	161
Go the ER/Hospital/UC	35%	95%	18	228
Call the doctor/make appointment	35%	85%	14	98
Use any non‐asthma medications (pain medicine, cough syrup, cold medicine)	47.5%	57.5%	30	54

Note

Instances = number of times a symptom or response was used in a card sort or mentioned by a participant during the course of the interview (excluding the interviewer's words).

Participants discussed using non‐pharmacologic symptom management strategies five times as often as using asthma medications (Table 2). Non‐pharmacologic strategies included: (1) getting a drink; (2) restricting activity; (3) breathing control; (4) calming down; and (5) waiting/toughing it out. Nearly half used alternative medications to relieve symptoms (pain, allergy, cough/cold medication and rubs). While most described experiences with medical care, few sought health care to help manage symptoms (35%; 14/40). Trigger avoidance was also uncommon (37.5%; 15/40 participants) as many triggers were considered difficult or impossible to avoid (ie job exposures, weather).

**Figure 2:**
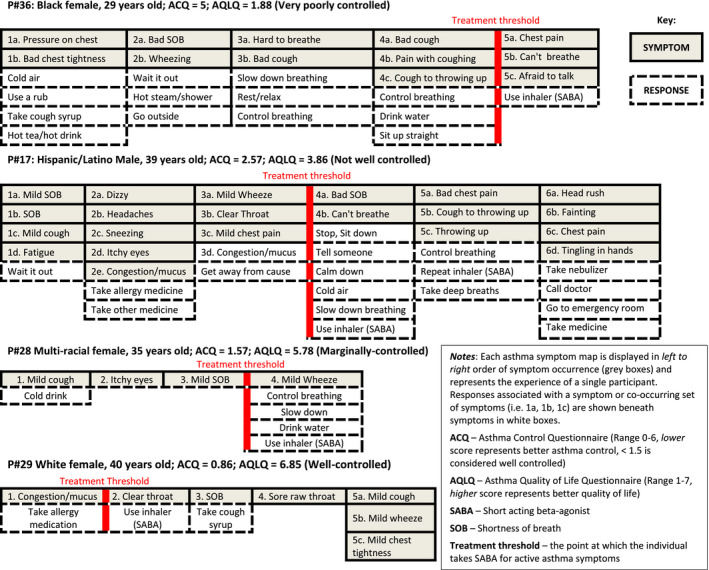
Comparison of symptoms, self‐management, asthma control, and quality of life between adults with well‐controlled, marginally controlled, not well‐controlled, and very poorly controlled asthma using card sorts

As seen in Figure 2, which depicts card sorts created by different participants, treatment thresholds (ie the point at which short‐acting beta‐agonists were used to treat active symptoms) were delayed in those with worse asthma control. Higher symptom frequency and worse asthma control were associated with greater use of non‐pharmacologic strategies (*r *= 0.645, *P* < .00; *r *= 0.360, *P* = .022, respectively). Patients who used a higher percentage of non‐pharmacologic strategies for symptom control also had lower FEV1_%pred_ (*r* = −0.341, *P* = .031). A moderate association was seen between emergency department use and asthma quality of life (*r *= 0.389; *P* = .013). No significant association was found between age and frequency of symptoms or self‐management responses.

### 3.1 Summary of qualitative interview themes

**Figure 3:**
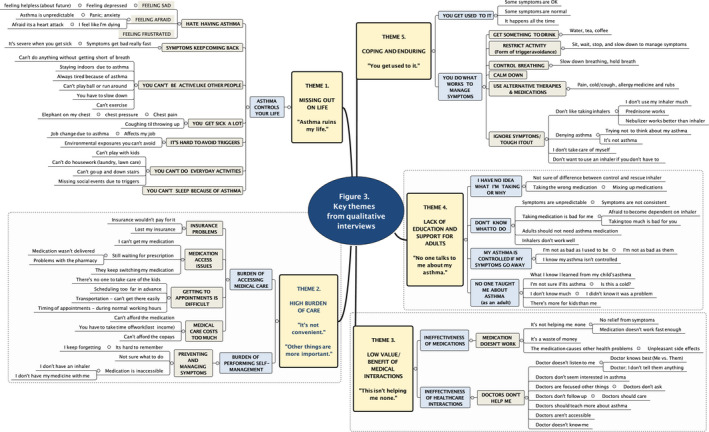
Key theme from qualitative interviews and supporting coding schema

Five themes were identified: (1) having asthma means being limited and missing out on things you want to do (*Missing out on life*); (2) health care for asthma is burdensome, and other things are more important (*High burden of medical care*); (3) there is not enough personal benefit from medical interactions to make preventive care worthwhile (*Low value/benefit of medical care*); (4) there is limited support and education about asthma for adults (*Insufficient education and support*); and (5) people normalize chronic symptoms over time, learn to "tough it out," and find ways of coping that fit with their lifestyle (*Coping and enduring*). The coding schema for these themes is displayed in Figure 3 with supporting data presented in Table 3.

**Table 3:** Main themes from qualitative interviews (N = 40) and supporting quotes

Theme 1: MISSING OUT ON LIFE. Having asthma means being limited and missing out on things you want to do	
Asthma causes people to miss out on: Family activities Playing with kids Social events Going outside Being active Doing housework Having fun	P2 (B, F, 41Y): [if] my breathing was better, I’d be able to do more things I enjoy. Like garden, yard work, you know, running around with the kids. Do them steps. P4 (B, F, 29Y): I had a friend that a wedding in the summer. [But] I couldn’t go because it was [hot] outside, and she was upset…how do you explain that to people? P5 (B, F, 28Y): It’s the part that I can’t breathe…I haven’t been active in sports or played basketball, did anything like that in a few years. P8 (W, F, 34Y): I like the outdoors and with asthma I barely can be out there. P9 (B, F, 34Y): I am very limited. Sometimes I don't like to go out because I get shortness of breath, I get dizzy. Grocery shopping and laundry, it's triggered me. P12 (B, F, 27Y): I just sit there because of my asthma. My cousins are like why don’t you come out? [They] wanna go out and do things, and I can’t. P18 (W, F, 38Y): Playing with the children and going to the beach and the park, [I can't] do too much of that. P22 (HL, F, 23Y): I’m an active person, I like to be outside, playing around. Yesterday, my family, they were outside playing and I’m sitting there like, I can’t do it. P24 (MR, F, 36Y): I’m limited with a lot of things because of my asthma P25 (HL, F, 44): And that kind of bothers me because then it’s like it puts [my kids] down. Like, ugh, mommy can’t finish. P26 (B, F, 40): I want to get out and enjoy my life, [but] I don’t feel good. ((Coughing)) I can't even laugh. P30 (MR, F, 41Y): Spring cleaning, straighten up the house, and I was out of breath and my chest was really killing me, so I had to sit down. P34 (B, F, 40Y): I’ll just stay home, 'cause I do so much, I can’t breathe, so I’ll just tell my daughter, oh well I can’t do it right now, or I’ll just sit at home [for] hours P36 (HL, F, 28Y): I have nieces running around and I can’t really chase after them or I don’t do anything because I’m not going to be able to catch my breathing P37 (B, F, 25Y): I can’t even do five jumping jacks without me having to like, get water or just sit down and take a rest
Theme 2: HIGH BURDEN OF ACCESSING CARE. Trying to manage asthma is burdensome and other things are often more important	
Taking care of asthma is complicated and difficult: It is too expensive Insurance is confusing Getting to office visits can be a problem Life is busy and chaotic Planning ahead is hard Other things are more important: Taking care of family Getting to work Paying the bills	P2 (B, F, 41Y): My transportation‐they work, too. And I have to go by their hours. P4 (B, F, 29Y): Being a mom with 4 kids, sometimes my health takes a back seat. P5 (B, F, 28Y): Time—lack of time… it’s too much time investment out of a schedule that already doesn’t work. My kids come first, and I’m working. P6 (B, F 26Y): I don’t have time to get up at 8am and come into your office. I need sleep…and [I got to] pay for this visit and pay for parking too. P10 (B, F, 30Y): I call [the pharmacy] and tell them I need a refill, they gotta send the doctor orders or something crazy … I’m so frustrated, I’m not calling back. P9 (B, F, 34Y) I was over the [Medicaid ] income requirement because I picked up two shifts… they made me skip a whole two months [medication]. P15 (W, M,39Y): Why do I have to make an appointment three weeks out? I’m never going to remember in three weeks that I have to go see you. P15 (W, M,39Y): Last inhaler that I had was almost a year ago…. I didn’t have the insurance P19 (B, F, 40Y): I don’t wanna take time off of work to go to the doctor because I miss out on my income. P23 (AS, F, 23Y): I’m not, like, poor but like, spending money on this inhaler, or medication, or doctor’s visits—I feel like it’s kind of a waste of money P28 (MR, M, 35Y): I’m behind on all my medicines 'cause they switched my pharmacy and I don’t have a car. Sometimes my meds will sit there and I can't get them. P31 (W, ,M, 39Y): I’m always working—my schedule is morning 'til evening. It’s hard for me to get to my doctor’s office. I got so much going on all the time, it’s insane. P34 (B, F, 40Y): I can’t afford they parking—they too expensive P38 (B, M, 30Y): I just want to breathe…but right now, I’m working 6 days a week, 9‐5, and there’s no doctors on Saturday… It’s mandatory that I work my job because that’s how I survive, but I still should be able to get healthcare…My jobs insurance ran out or something like that and I didn’t even know. P39 (W, F, 24Y): Rotating schedules at work, I never know when I’m going to be awake. If I’m supposed to take it morning and night, chances are that’s not happening
Theme 3: LOW VALUE/BENEFIT OF MEDICAL CARE. Nothing seems to help …so it isn't really worth the effort	
Medications do not seem like they work well. Inhalers: Fail to relieve symptoms Work too slowly Have side‐effects Do not have enough benefit to make it work taking Are hard to remember to take	P3 (W, M, 37Y): Should be working, but it doesn’t…You say, I shouldn’t be using my inhaler that much. I say, I can’t breathe. P10 (B, F, 30Y): I feel like the inhalers don’t work… [and] I hate the way my mouth feels so dry—that’s the worst. I rather use a different remedy versus that inhaler. P13 (MR, F, 39Y): I feel like the medications don’t work… it’s a little frustrating, you know. P14 (B, F, 25Y): They tell you if you take [albuterol] too much then you shouldn’t be taking it. And I have to take it 6 times for me to even feel like it’s starting to work. P15 (W, M,39Y): That's one they always hand me for control. I’ve never seen a difference what so ever…so, it gets tossed to the side [because] it does not work. P17 (HL, M, 38Y): I don’t see the relief so my instinct is don’t take it, [or] I don’t feel relief so I end up taking more and it’s not necessarily the best course of action. P21 (B, F 40Y): I try to see if that’s gonna work, and take like maybe 8 or 9 puffs. That don’t work too very well. P25 (HL, F, 44): I won’t even take it, cause it’s not doing nothing for me. [My daughter] she's like “well just take it anyways.” So I’ll take it [but] I’m still short of breath. P26 (B, F, 40Y): They say to take it twice a day, but twice a day is not good enough. [I need] 6 puffs of each one a day. P30 (MR, F, 41Y): It eases up a little bit, but it seems like it takes a couple days to go away…it will still linger around P31 (W, ,M, 39Y): it’s just it’s too hard to remember it every day. You know it’s what it boils down to. P34 (B, F, 40Y): I told them, it don’t work, and he didn’t even ask if we could change it. [so] I don’t use it as much because [it] doesn’t work, so there’s no point. P36 (HL, F, 28Y): I hit my inhaler once, twice and [in] 45 minutes I got to hit it again…Refill doesn’t do anything. You can keep that. I’m telling you, it doesn’t work. P37 (B, F, 25Y): Honestly, nothing controls my symptoms….. I’ll take my rescue inhaler about 4 times a day…I’ll be like, “Ugh, this thing isn’t gonna work anyways.
Healthcare providers do not seem like they help. HCP often don't: Really listen (40%) Know the patient (37%) Focus on the patients' priorities and needs Seem caring and connected Follow up on concerns and treatment plans Ask questions about asthma specifically	P1 (B, M, 26Y): I feel like I just go see doctors. I don’t feel like they actually try to fix it or figure out. It’s just ‘okay here’s this medicine. … It’s not helping me none. P3 (W, M, 37Y): I got tired of asking for refills because my doctor looks at me like, “hey you’re not supposed to run through that much.” P4 (B, F, 29Y): My asthma doesn’t seem like it’s a priority with my doctors…I just feel like it’s something on the list to check off and, “Boom, you’re out.” I pulled back from going to the doctor so much because I felt like it wasn’t helping enough…I’ll just take care of it myself. P8 (W, F, 34Y): The doctors are so busy, we don’t really get to talk [about asthma] unless it’s after my ER visit. Other than that, they don’t really talk to me about it. P13 (MR, F, 39Y): Maybe they should not focus on being so weight‐based…they want you to lose weight [and] I’m working on it, but it hasn’t improved the symptoms. P14 (B, F, 25Y): As a kid, they told me how I should be taking my medicine, but I didn’t really follow. [Now]They just tell me “you just got to stop smoking" P19 (B, F, 40Y): I had to call every day to see if they sent the inhaler over…then again when I went to go pick up my meds, it still wasn’t in there….I just said forget it. P26 (B, F, 40Y): [I said] The medicine’s not working. And he said “make sure to get it, take it on time.” I said, I do that every day and it’s still like this. [He said] just to give it more time. What do you mean more time? I’ve seen you 3 months ago! P22 (HL, F, 23YY): I will speak up like, hey my asthma is been getting a little bad, and they just keep telling me that my asthma is ok, it’s not that much of a problem. P28 (MR, M, 35Y): They haven’t paid a lot of attention… if I say anything about my asthma, it’s just do I have my inhalers? Yes. Are you using them? When I need to. P36 (HL, F, 28Y): No one asks about my asthma. They just focus on my mental health. It’s robotic. Same questions, same order… they don’t do [asthma] follow‐ups P37 (B, F, 25Y): I feel like I’m not connected with my doctor…. just my own personal thing [so] I go to the emergency room. P39 (W, F, 24Y): I don’t know [my doctor]. You want someone who’s gonna follow you and understand. You don’t want to have to explain everything, every time. P40 (HL, F, 30Y): I have asthma for almost my whole life are you telling me I can’t get a fucking inhaler? (crying) I have to prove it to you? It don’t feel like they listen.
Theme 4: LACK OF EDUCATION/SUPPORT OF ADULTS. As an adult, no one teaches you about managing asthma, so you're not sure what you're doing.	
Teach patients how to manage asthma	P2 (B, F, 41Y): Control inhaler? I don’t even know what that is. P3 (W, M, 37Y): I haven't been taught about asthma. I’m a clean slate…in 30 years, I've been taught nothing. P7 (W, M, 29Y): Honestly, I haven't been taught anything.… I’ve just been doing what I’m told, just use my inhalers and if it gets worse just to go to the hospital P9 (B, F, 34Y) I don't know much, like, besides like your lungs inflaming. I haven't really been taught anything about how to try to prevent attacks and stuff like that. P10 (B, F, 30Y): I know my lungs can really shut down or something but I didn’t really learn too much about asthma. P12 (B, F, 27Y): A rescue inhaler is something that holds you off probably until you make it to the hospital. P15 (W, M,39Y): A lot of it has to do with anxiety—I wasn’t sure if I actually have to do [the inhaler] P16 (HL, F, 24Y): I’ve been taught it’s like a chronic disease… you start wheezing, it means that your lungs are swollen and you retain fluid in your lungs. That’s about it. P17 (HL, M, 38Y): I’ve never been well‐informed about asthma and how it relates to me and what it means to me. P19 (B, F, 40Y): They never taught me anything about it, [and] I’ve never asked about it. P26 (B, F, 40Y): I just know about my inhaler, that’s it…I don’t really know anything. P30 (MR, F, 41Y): (Laughs) I’m not sure of the difference [between a control and rescue inhaler]. My biggest challenge is when to know to use it, and how much. They have more for the kids than they do for me…I’m just kinda confused… It’s been a long time since I actually sat down with my doctor and discussed asthma. P31 (W, ,M, 39Y): They said if you don’t take it every day, there’s no point in doing it…. if you miss a day here, it’s the same thing as not taking it to begin with. P33 (B, F, 32Y): Your air way will close, that’s pretty much what I do know about asthma P40 (HL, F, 30Y): All they do is give you a big ass pamphlet to read. Nobody even understands that shit. Like I don’t know what the hell that means, all those words.
Theme 5: COPING AND ENDURING. Over time, you get used to having symptoms. You learn to tough it out, and find other ways of coping.	
	P2 (B, F, 41Y): When I start wheezing, I just usually just sit down and do my deep breathing exercises. Try and relax. Try not to think about it. Coping mechanisms to help you get not over anxious – try to slow you down. I don’t get rest because I’m up coughing at night. I don’t know the last time I had a good night’s sleep. P5 (B, F, 28Y): It’s not supposed to be normal, but for me pain is normal….I drink water, control my breathing, and tough it out. I learned to live with it P8 (W, F, 34Y): You know this daily stuff [symptoms], I’ve gotten used to it—It doesn’t really scare me. I’ve been dealing with it so many years that I’m just used to it. P10 (B, F, 30Y): I’m rushing to get out of there [doctors’ office] and if I’m not having issues going on today…then I wouldn’t say nothing… P14 (B, F, 25Y): Sometimes I [take the inhaler] but I'm used to waiting it out… I don’t think about it till it’s too late and I’m already in the bad stage of it. P15 (W, M,39Y): Most of the time I tough it out [because] it cost money. I tough it out until it goes away, I have no choice—I got to work and I don’t have an inhaler. P16 (HL, F, 24Y): I usually just try to stick it out, and wait until it gets better…when the wheezing starts I’ll try to tough it out and see if I could go without medications. P17 (HL, M, 38Y): This is something I’ve always lived with, never really tried to manage or treat. I usually take [the inhaler] only when my symptoms are really severe. P19 (B, F, 40Y): Sometimes I just tough it out… Put it onto the back burner, brush it off [until] I’m coughing till I’m about to throw up… P22 (HL, F, 23Y): Tough it out…Take a deep breath. Wait it out… let’s try to tough it out, control my breathing on my own. P23 (AS, F, 23Y): Tough it out, tough it out… I do that with everything. I feel like I should be breathing more but I’m just so used to this that it’s normal for me. P24 (MR, F, 36Y): I’ll wait to take my pump until last. And the reason I do that is because I want to try my hardest to get through it without steroids.

Note

Participant quotes are identified by participant number (P#), followed by (Race, Sex, Age in years). *Race:* As = Asian, B = Black, HL = Hispanic/Latino, MR‐multiracial, W = White. *Sex*: F = female, M = male.

### 3.2 Theme 1: Missing out on life

Young adults believed that having asthma meant living with symptoms and being unable to do as much as people without asthma. All expressed a strong sense of missing out on life and being limited in the activities they wanted to do. This included decreased ability to be physically active, go out with friends, play with their children, attend important social events, and perform common activities of living, such as walking upstairs, doing laundry, grocery shopping, and household activities. The impact of uncontrolled asthma on daily life is evident in the following quotes:

P1: I get frustrated, especially when I’m out for a good time and everybody’s sitting there laughing and I’m over here, choking because I can’t breathe. (Black, male, age 26)

P13: I would definitely go out more if I could, you know, breathe. I used to go to the park and walk around—we don’t do any of that stuff anymore. (Multiracial, female, age 39)

### 3.3 Theme 2: High burden of medical care

While feeling that asthma substantially limited activities and quality of life, participants indicated that trying to manage asthma was too difficult, specifically with regard to accessing medications and medical care. Many indicated their life was busy and chaotic. Finding time to get to appointments required too much time and effort, and often conflicted with other personal needs. A recurrent challenge was scheduling and keeping appointments, as office hours occurred during the working week, which required taking time off work to get to visits. As one woman explained:

P37: It’s too much effort. What’s it doing for me? I only get ten personal days which are maxed out. After that, I’m gonna lose money and I’ve gotta take care of home. My asthma doesn’t seem like a priority with my doctors, so I feel like, “Why am I gonna stress myself out to go?" (Black, female, age 25)

Many indicated taking care of kids/family, getting to work, and paying the bills was more important than managing asthma. Because medical management conflicted with these priorities, it was often deferred until urgent care was required.

P10: It just doesn’t work for me. I don’t make the time to take care of my asthma because I’m always so tired and busy, and my schedule is hectic…If it’s not bothering me at the time, I don’t mention [asthma] at all…unless it's to the point where I can’t breathe….(Black, female, age 30)

Difficulties with insurance (lapses, medications not approved by plan) and financial challenges were another source of burden, including cost of medications, co‐pays, parking fees, transportation costs, and lost income.

### 3.4 Theme 3: Low value/benefit of medical care

A dominant theme throughout interviews was that there was not enough benefit from asthma‐related preventive care to make it worth the effort of engaging. Many indicated the medical care they received in the past had not helped them much. Combined with Theme 2, this translated to a high burden of accessing care with little perceived personal benefit:

P4: I kind of pulled myself back from going to the doctor because I felt like it wasn’t helping enough. I felt like okay….I’ll just take care of it myself. (Black, female, age 29)

P6: I feel like the cost benefit ratio is not fair going to my primary care. If I have to go anyways, then I’ll talk to them about it. But if it’s specifically for my asthma, I’m not doing that. (Black, female, age 26)

Lack of benefit occurred in two main areas: (1) medications do not work well, and (2) healthcare interactions do not help much.

#### 3.4.1 Medications do not work

Of forty participants, nine had no rescue inhaler (22.5%). Of those who did, 83.8% (26/31) indicated that they often avoided taking it because they felt the medication either did not work at all or did not work fast enough. As one man commented, *"I don't see the relief so my instinct is don't take it" (P17, Hispanic/Latino, male, age 37)*. Consequently, participants often used non‐pharmacological methods of managing symptoms (eg activity restriction, breathing control) instead of prescribed therapy to relieve active symptoms (92.5%; 37/40 participants). Many were unaware of differences between control and rescue medication (50%; 20/40). For example, "*I use a blue one and a red one…they're both the exact same thing" (P7, White, male, age 29),* and "*Control inhaler? I don't even know what that is…*" *(P2, Black, female, age 41)*. Among those aware of being prescribed control medication, similar beliefs regarding ineffectiveness were voiced as a rationale for not using:

P15: That's one they hand me for control. I’ve never seen a difference what so ever…so, it gets tossed to the side [because] it does not work. (White, male, age 39)

#### 3.4.2 Healthcare interactions do not help

In interviews, nearly 80% (32/40) of participants expressed frustration regarding their asthma‐related health care. On survey, 50% (20/40) indicated dissatisfaction with their asthma care, and 52.5% (21/40) reported feeling that healthcare providers (HCP; commonly referred to as "doctor") did not help when it came to managing asthma. Greater satisfaction with care was associated with better asthma control (*r *= 0.411, *P* = .008) and marginally associated with quality of life (*r *= 0.30, *P*=.058). As seen in Table 3, this included recurrent sub‐themes that the HCP did not listen to and prioritize patient concerns, did not seem caring or connected, and did not follow up on treatment plans to evaluate for effectiveness. In particular, patients disliked seeing different HCPs, as this limited ability to develop a relationship and required them to repeat information at visits. Furthermore, many felt HCPs did not view asthma as a priority and were focused on other health and psychiatric issues to the exclusion of asthma (eg mental health, comorbidities, obesity). Participants indicated that HCPs rarely asked specific questions about asthma. For example:

P1: When I go the doctor, they ask about all my other stuff, but they never ask about my asthma (Black, male, age 26)

P37: They usually ask something like, “have you had trouble breathing in the last year?” Real quick like it isn’t that important … I say “it’s OK,” because it is fine in the moment and I can’t go back deeply like that on the spot. (Black, female, age 25)

Others reported feeling rushed during visits and felt the doctor did not give adequate attention to their concerns that medications were not working or that they had trouble obtaining medications.

P7: I don’t feel like they listen. They be there for five minutes and then you’re done…They just keep giving me different medications…Why? (White, male, age 29)

P8: The doctors are so busy, we don’t really get to talk [about asthma]. (White, female, age 34)

### 3.5 Theme 4: Insufficient education and support for adults with asthma

In addition to the high burden of medical care and perceived lack of benefit, participants indicated that they did not receive support or education for managing asthma as an adult. As one man commented, "*I don't see a lot of doctors explaining to people what asthma is, how it can affect you." It's like “Hey you got asthma, take an inhaler" (P17, Hispanic/Latino male, age 37)*. Some recalled receiving asthma education as paediatric patients but indicated they were unable to recall what they learned during childhood. Consequently, participants felt they did not know enough as adults to manage asthma effectively. Several specifically noted that the withdrawal of paediatric supports made it harder to control their asthma as adults. For instance:

P10: [as a kid] the doctor explained it to me. I was good back then, I was controlled. But I had help you know? I had parents who stayed on me, I went to school and my nurse stayed on me… (Black, female, age 30)

P37: coming from pediatrics to my primary care doctor, no one ever told me anything about asthma, so I’m just going based off of what my mom told me. I don’t really know a lot besides you have a problem breathing. That’s all I know. (Black, female, age 25)

The two most commonly identified areas of concern to participants were lack of understanding about managing asthma in general (80%, 32/40) and asthma medications (65%; 26/40). This included knowledge about which medication to use, how much to use, when to use it, side effects, and expected onset of symptom relief. This interview finding was substantiated by surveys: 87.5% of participants thought all inhalers worked immediately, and 62.5% (25/40) believed overusing inhalers could lead to addiction.

Understanding of asthma control was poor. Most participants thought asthma was uncontrolled *only* if symptoms occurred multiple times daily (35%; 14/40), did not resolve with SABA use (25; 10/40), occurred more often than usual (17.5%; 8/40), or they could not figure out how to handle symptoms (15%; 7/40). Most (80%) believed that the primary purpose of controlling asthma was to decrease medication use, prevent dependency, and avoid complications from overusing medication, which contributed to decisions to minimize medication use even when symptomatic. As one woman expressed:

P12: I stopped because I was addicted to always doing [the inhaler] if I wheezed. (Black, female, age 27)

Even those who felt knowledgeable (12.5%; 5/40) had poor understanding of effective self‐management. This is illustrated in the following quote:

P21: I’ve been taught a lot of stuff about asthma… they say you can control it by using your pumps and steam in the bathroom. I grab this [inhaler] and take like 8 or 9 pulls, that don’t work … I sit in the bathroom 4 or 5 hours in the steam. (Black, female, age 29)

### 3.6 Theme 5: Coping and enduring

The large majority of participants had daily symptoms (87.5%; 35/40 participants), and more than half reported experiencing anxiety or panic related to symptoms (21/40). Symptoms that occurred on a regular basis were normalized over time (100%; 40/40 participants) as seen here: "*I guess I’ve just been living with it, so it's normal" (P6, Black, female, age 26)*. Furthermore, as ability to tolerate symptoms increased over time, use of medication correspondingly decreased. Instead of using medication, many waited for symptoms to resolve and tried to “tough it out” or held off on taking medications as long as possible (65%; 26/40 participants).

P1: I’m used to wheezing. I go through this all the time, so I don’t always take [the inhaler]. (Black, male, age 26)

Many participants preferred using non‐pharmacologic symptom management strategies (87.5%), as this helped to reduce medication use and increased their sense of control over asthma. Medications were typically reserved for bothersome symptoms with greater than usual severity. Participants often did not use inhalers for "normal" symptoms that could be managed by other means, such as restricting activity to reduce symptom burden.

P20: I try not to use my medicine. My doctor he told me over using it ain’t good. I don’t wanna get immune to it. (Black, male, age 37)

P31: I ignore it until I can’t handle it. I keep my normal daily activities to a minimum at a regular basis, so I don’t need [the inhaler.] (Caucasian, male, age 39)

## 4 DISCUSSION

Our findings show the extensive and pervasive burden of disease experienced by young adults living with uncontrolled asthma. Many participants in this study experienced debilitating daily symptoms for which they chose *not* to use quick‐relief medication or seek medical attention due to the difficulty of accessing care *and* the limited perceived benefit of prior healthcare interactions and medications. Instead, non‐pharmacologic symptom management strategies were often used to control symptoms (eg restricting activity). Thus, even though asthma "ruined" their quality of life and limited ability to do everyday activities, many found enduring symptoms to be *less* burdensome than engaging in preventive healthcare.

This highlights a substantial healthcare problem that is applicable to a range of chronic diseases. How can we expect to increase engagement in preventive care if the perceived burden of accessing care is *greater* than the burden of uncontrolled disease? In order to improve individuals' self‐management and population health outcomes, we must recognize that the *burden of disease* not only encompasses the effect of illness on health and quality of life, but also the *burden of disease management*—specifically, the enormous effort required to manage a complex illness effectively in a non‐user‐friendly healthcare environment. Young adults may be particularly at risk in this regard, as transition to adulthood and autonomous self‐care is often accompanied by loss of paediatric support services and an established primary care provider.^14^, ^45^, ^46^ While there are no data on the frequency of preventive asthma visits in young adults,^4^ other chronic disease literature indicates preventive care may be half that of adults over age 45, thus explaining elevated rates of emergency care utilization and declining medication adherence in this population.^47^

Ironically, the low personal value of healthcare interactions for young adults with asthma could be partially attributable to "value‐based" approaches to care, which require HCPs to accomplish numerous preventive tasks within the short time allotted for a standard visit (eg smoking cessation counselling, depression screening). Thus, the disturbing observation by participants that HCPs were often preoccupied with other issues holds strong face validity. While health maintenance items are important from a population health perspective, it is imperative to recognize that such agendas may not correspond with patients' views of their healthcare needs and that in prioritizing population health we risk marginalizing and alienating individuals. This raises the ethical question: Whose values should be prioritized in value‐based health care?

It is almost certain that improving outcomes will require modifying current approaches to preventive care, including minimizing barriers (eg making care convenient) and maximizing benefits (making care meaningful and effective from patient perspectives). This might include more aggressive treatment and follow‐up to ensure that asthma medications (quick relief *and* controller) are being used at the proper dose and technique to quickly and effectively reduce symptoms. Additionally, greater intentionality on the part of clinicians might be needed to reengage patients who have been alienated by prior experiences, as these individuals might not report symptoms. Ultimately, changing outcomes will entail carving out time to systematically assess and educate adult patients about asthma, or devising alternate care models that can address critical gaps in care.

Lastly, our findings suggest that young adults normalize regularly occurring symptoms and learn to tolerate progressively greater symptom severity over time. Clinician training may be needed to increase awareness and to promote accuracy of clinical assessments. Asking about specific symptoms along with symptom management strategies could help identify those who are not well controlled, as greater numbers of non‐pharmacologic strategies suggest higher levels of uncontrolled symptoms.^15^, ^48^ It is also worth observing that coughing was the most commonly mentioned symptom, with many coughing to the point of vomiting. This finding, similar to adolescent populations, suggests that coughing may be a particularly bothersome symptoms of asthma from patients' perspectives.^35^, ^36^ Clinicians may want to monitor for the presence of asthma‐related coughing and educate patients how controller medication can reduce coughing to promote adherence. Lastly, it may be useful to consider word choices when assessing symptoms. While "chest tightness" is the accepted clinical term, "chest pain" and "chest pressure" may be more reflective of the patient experience. Incorporating patient‐centric terminology validates individuals' experiences and might be useful in developing therapeutic relationships.

### 4.1 Limitations

Participants in this study were predominantly lower socio‐economic status, young, urban adults from a hospital‐based primary care clinic that had higher rates of uncontrolled asthma than the general US population (61.9% vs. 82.5%).^1^ Findings may not be generalizable to non‐equivalent populations or may only be reflective of similar patients with uncontrolled asthma. Additionally, data were collected at single time‐point from a small sample of developmentally diverse adults (emerging and midlife), and distinctions between age groups and changes over time were not identified. Repetition in a more diverse sample with attention to age‐related changes in self‐management may be warranted. Nonetheless, our findings indicate concerning patterns of suboptimal asthma management in at‐risk young adults, and highlight the urgent need to improve clinical assessment and asthma management, as well as avenues for future research.

## 5 CONCLUSIONS

Young adults with uncontrolled asthma may normalize symptoms over time and elect to use non‐pharmacologic symptom management strategies instead of using asthma medications or seeking preventive care that could lead to controller medication. Living with recurrent symptoms may be viewed as less burdensome than engaging in preventive health care. Enhanced training for clinicians in patient‐centric asthma care may be needed to achieve meaningful change in outcomes for patients.

## CONFLICT OF INTEREST

The authors have no conflict of interest to declare.

## AUTHOR CONTRIBUTIONS

Jennifer R. Mammen and Judith D. Schoonmaker contributed to data collection, analysis, interpretation, and manuscript preparation. Kelsey Turgeon, Ashley Philibert, and James Java contributed to data analysis and interpretation. Jill Halterman, Marc N. Berliant, Amber Crowley, Marina Reznik, Jonathan M. Feldman, Robert J. Fortuna, and Kimberly Arcoleo contributed to results interpretation, manuscript preparation, and revisions.

## Data Availability

The data that support the findings of this study are available from the corresponding author upon reasonable request.
